# DC Respond to Cognate T Cell Interaction in the Antigen-Challenged Lymph Node

**DOI:** 10.3389/fimmu.2019.00863

**Published:** 2019-04-25

**Authors:** Caterina Curato, Biana Bernshtein, Eva Zupancič, Almut Dufner, Diego Jaitin, Amir Giladi, Eyal David, Louise Chappell-Maor, Dena Leshkowitz, Klaus-Peter Knobeloch, Ido Amit, Helena F. Florindo, Steffen Jung

**Affiliations:** ^1^Department of Immunology, Weizmann Institute of Science, Rehovot, Israel; ^2^Research Institute for Medicines (iMed.ULisboa), Faculty of Pharmacy, University of Lisbon, Lisbon, Portugal; ^3^Medical Faculty, Institute for Neuropathology, University Freiburg, Freiburg, Germany; ^4^Life Science Core Facilities, Weizmann Institute of Science, Rehovot, Israel

**Keywords:** DC, T cell, cognate interaction, immune synapsis, RNAseq

## Abstract

Dendritic cells (DC) are unrivaled in their potential to prime naive T cells by presenting antigen and providing costimulation. DC are furthermore believed to decode antigen context by virtue of pattern recognition receptors and to polarize T cells through cytokine secretion toward distinct effector functions. Diverse polarized T helper (T_H_) cells have been explored in great detail. In contrast, studies of instructing DC have to date largely been restricted to *in vitro* settings or adoptively transferred DC. Here we report efforts to unravel the DC response to cognate T cell encounter in antigen-challenged lymph nodes (LN). Mice engrafted with antigen-specific T cells were immunized with nanoparticles (NP) entrapping adjuvants and absorbed with antigen to study the immediate DC response to T cell encounter using bulk and single cell RNA-seq profiling. NP induced robust antigen-specific T_H_1 cell responses with minimal bystander activation. Fluorescent-labeled NP allowed identification of antigen-carrying DC and focus on transcriptional changes in DC that encounter T cells. Our results support the existence of a bi-directional crosstalk between DC and T cells that promotes T_H_1 responses, including involvement of the ubiquitin-like molecule Isg15 that merits further study.

## Introduction

T cell encounter of major histocompatibility complex peptide (MHCp) entities on dendritic cells (DC) has, depending on its context distinct outcomes. Protective T cell responses include proliferation, T helper (T_H_) cell polarization, and memory formation. They are believed to rely on three distinct stimuli: cognate MHCp encounter, costimulatory signals provided by B7 family members, and instructing cytokines. All three signals can be derived from DC for productive T cell priming to occur (1). Furthermore, the stimuli seem to have to come from the same antigen-presenting cell (APC), because only pathogen-exposed DC can direct full T_H_ cell differentiation (2). Recognition of pathogen-association of antigen by DC, therefore, seems critical for the initiation of protective T cell responses, suggesting that inflammatory mediators can amplify, but not initiate, adaptive immunity. Such a scenario ensures that information on the original context of the antigen, which is deciphered by DC through pattern recognition receptors (PRR), including Toll-like receptors (TLR) (3), can be relayed to T cells. DC populate lymphoid organs, i.e., spleen and lymph nodes (LNs), as well as peripheral non-lymphoid tissues, such as skin, intestine, and lung. From the latter, DC traffic via the lymphatics to the LNs, where antigen-loaded immigrant and resident DC encounter naïve T cells to initiate adaptive T cell immunity (4, 5).

Classical DC (cDC) comprise two main subsets, termed cDC1 and cDC2 (6–9).

XCR1^+^ cDC1 are specialized in the stimulation of CD8^+^ T cells (10–12). XCR1^+^ cDC1, often also defined as CD8α^+^ or CD103^+^ DC (13–15), depend on the transcription factor Batf3 for their development (16). cDC1 are superior in cross-presentation of cell-associated antigens and provide a critical source for IL-12 during infection (15–18). CD11b^+^ cDC2 that are further defined by SIRPα (CD172A) surface expression, depend for their development on the transcription factors Irf4, Klf4, and Notch2 (19–23). Functional specialization of cDC2 is less well-understood, although these cells were reported to be superior in MHC-II presentation, and hence the stimulation of CD4^+^ T cells (24).

Once polarized, T_H_ cells have acquired effector functions to combat infections and malignancies and contribute to the immune defense. Aside from regulatory T cells (T_reg_), CD4^+^ T cells can differentiate into diverse helper T_H_ lineages, e.g., T_H_1, T_H_2, T_H_17 cells, that are characterized by distinct effector cytokine profiles and specialized in control of viral, bacteria and fungal pathogens. CD8^+^ T lymphocytes (CTL) acquire cytotoxic activity and are critical players in the defense against in intracellular pathogens (25).

It remains unclear if DC subsets harbor distinct potential for T cell polarization toward T_H_ fates, for instance as result of distinct TLR repertoires. XCR1^+^ cDC1 are required for protective CTL, T_H_1, and T_H_17 responses against bacterial and viral infections, as well as tumors (15–18, 26, 27), which might in part be due to superior efficiency in cross-presentation. Impairment of CD11b^+^ cDC2 development or function, through Irf4, Klf4 or Notch2 deficiencies, on the other hand, was reported to compromise T_H_2 differentiation, as well as the homeostatic generation of T_H_17 cells in mucosal tissues (19–23). In mice exposed to *Nippostrongylus brasiliensis* and the contact sensitizer di-butyl phthalate, CD11b^+^ and double negative skin DC transcriptomes differ from the respective non-treated controls but they share minimal transcriptional similarities though the induction of the same T_H_2 response (28).

In the DC/T cell synapse, DC trigger the T cell receptor (TCR) with MHCp and provide costimulation via CD80 and CD86. Whether the interactions with cognate T cells in turn license the DC to acquire polarization potential remains unclear. Here, we designed an experimental set up to probe for such putative DC responses to cognate T cell encounter in antigen draining LNs. Specifically, we immunized mice that had been engrafted with antigen-specific T cells (OT-I, OT-II), with nanoparticles (NP) entrapping antigen (OVA), adjuvants (CpG), and a fluorescent dye (6G rhodamine) to study the immediate DC response to T cell encounter using bulk and single cell RNA-seq profiling. Our results suggest the existence of a bi-directional crosstalk between DC and T cells to promote T_H_1 response that merit further exploration.

## Results

### Targeting Dendritic Cells by Antigen-Loaded Nanoparticles (NP)

**Figure 1:**
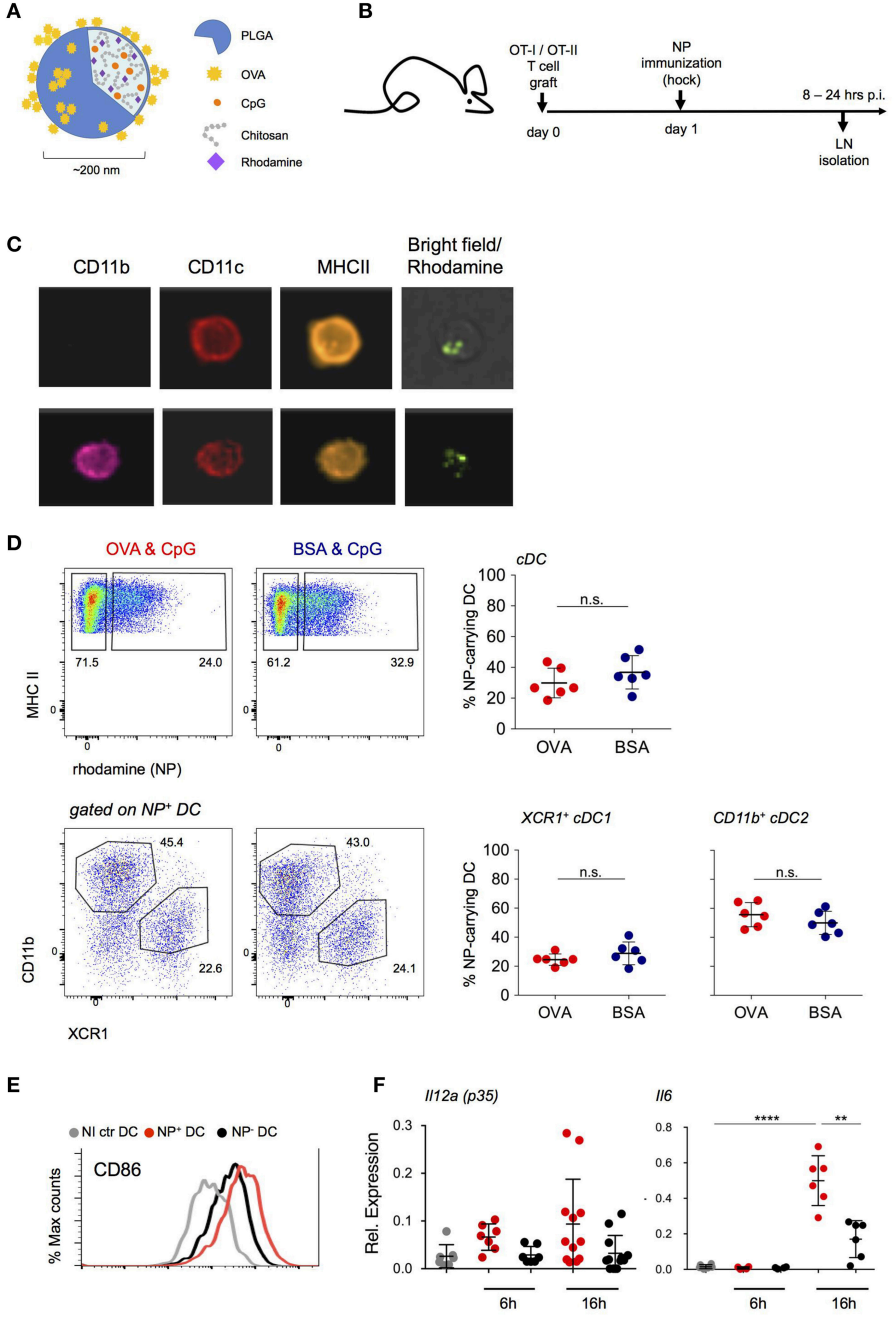
NP uptake correlates with an activated DC phenotype. **(A)** Schematic representation of the PLGA-based nanoparticles (NP) entrapping adjuvant (CpG ODN 1826) and fluorescent dye (Rhodamine). Antigen (OVA) was adsorbed onto the external surface. **(B)** Scheme of the experimental lineup for *in vivo* analysis of lymph node DC following s.c. immunization with NP. **(C)** *In vivo* internalization of NP by lymph node (LN) DC 16 h post immunization (p.i.) shown by ImageStream analysis. CD11c, CD11b, MHC-II staining and overlay of bright and rhodamine channels are shown. **(D)** Uptake of (OVA/CpG) and (BSA/CpG) NP by DC (upper, gated as CD45^+^ lin^−^ MHC-II^+^) and by cDC subsets, XCR1^+^ cDC1 and CD11b^+^ cDC2 (lower). *n* = 6, individual mice. **(E)** Surface expression of CD86 co-stimulatory marker in NP-carrying DC (NP+ DC, red), non-NP DC (NP- DC, black) and non-immunized DC (NI ctr, gray) 24 h p.i. **(F)** *Il12a(p35)* and *Il6* mRNA expression levels were measured by real-time PCR. NP+ DC, NP- DC and NI ctr DC were sorted from skin-draining LN 6 and 16 h p.i. Each dot represents a pool of 2 mice, *n* = 6–12, line represents mean ± SD. ^**^p ≤ 0.01, ^****^p ≤ 0.0001.

To define and isolate antigen-presenting DC from LNs of immunized mice, we employed targeted delivery of designed polymeric aliphatic-polyester poly(lactic-co-glycolic acid) (PLGA) nanoparticles (NP) (29). In their internal phase, these NP were engineered to entrap the fluorescent dye rhodamine 6G for detection and visualization and the TLR9 ligand CpG (ODN 1826) as adjuvant. CpG triggers *in vitro* and *in vivo* maturation of DC with redistribution of DC to the T cell zone in lymphoid organs, upregulation of MHC-II and costimulatory markers, as well as IL-12, IL-6, and TNFα production that promotes the development of T_H_1 responses (30–32). As antigen, Ovalbumin (OVA) was adsorbed onto the NP surface (Figure 1A). One day prior to subcutaneous (s.c.) hock immunization with NP, mice were engrafted with OVA-specific CD4^+^ or CD8^+^ TCR transgenic cells (Figure 1B). At defined time intervals shortly after immunization, inguinal and popliteal LNs of challenged mice were isolated for analysis by ImageStream and flow cytometry. NP were found associated with DC and other cells, including B cells, plasmacytoid DC, as well as non-phagocytic cells, such as T cells, albeit in different amounts (Supplementary Figure 1A, data not shown). LN DC that had engulfed NP could be readily visualized by ImageStream and included cDC1 and cDC2 as discriminated by CD11b expression (Figure 1C). NP^+^ DC comprised XCR1^+^ cDC1 and CD11b^+^ cDC2 in a reproducible ratio that was irrespective of the specific protein cargo (Figure 1D) and mirrored the abundance of the subsets in the non-immunized LNs (Supplementary Figure 1B). Phenotypic characterization of the sorted NP^+^ DC fraction revealed upregulation of CD86 surface expression as compared to DC isolated from the contra-lateral non-immunized LNs. The latter was more pronounced than that of NP^−^ DC of the same LN (Figure 1E). In comparison to NP^−^ DC, NP^+^ DC also displayed increased expression of *Il12a (p35)* and *Il6* mRNA, both 6 and 16 h post-immunization (p.i.) (Figure 1F). Collectively, these data establish the efficiency of NP-mediated antigen delivery to LN DC and show that NP internalization correlates with DC activation.

### NP Immunization Induces an Antigen-Specific T Cell Response and Polarization

**Figure 2:**
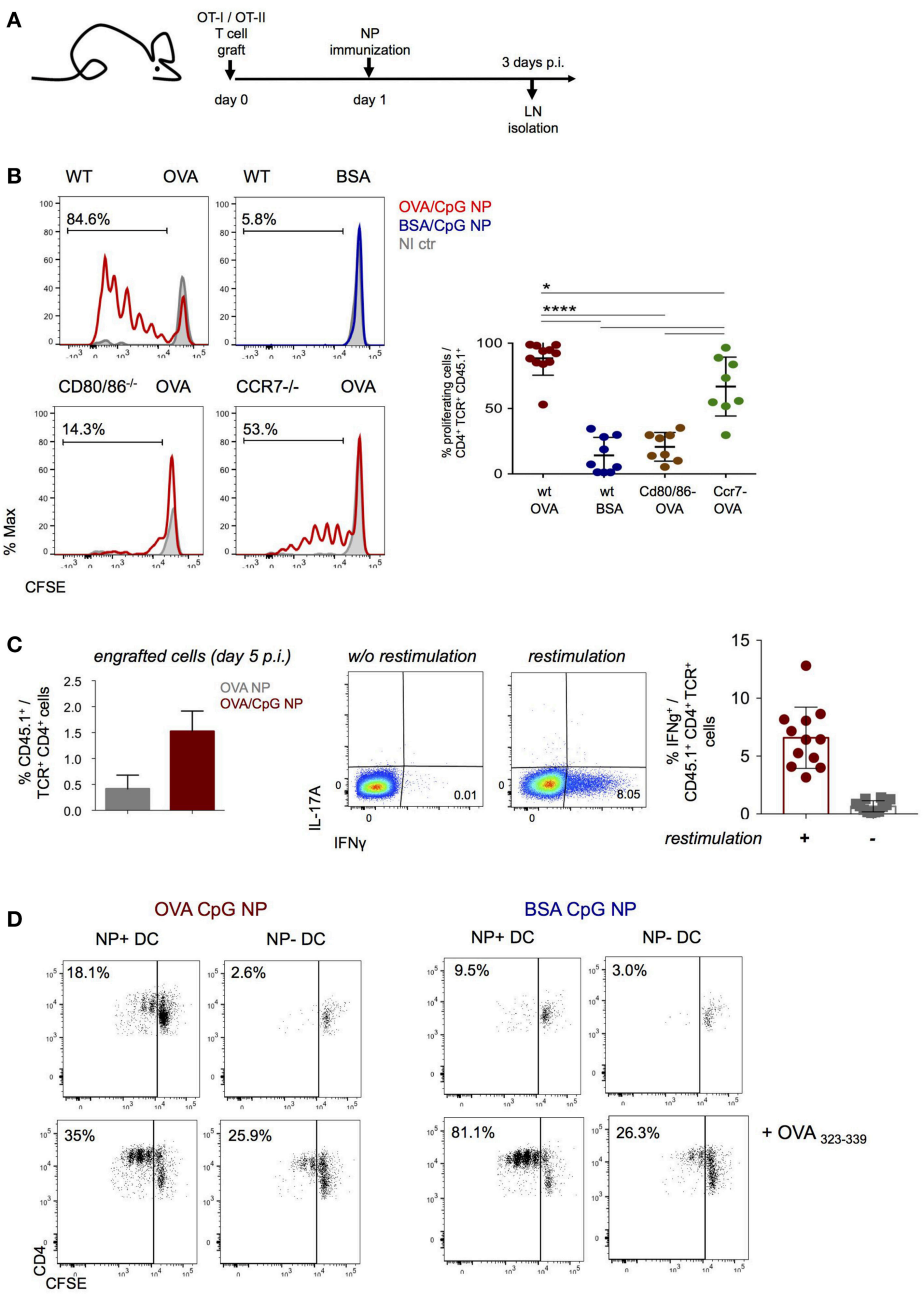
NP immunization induces an antigen-specific Th1 cell response. **(A,B)** CFSE-labeled CD4+ OT-II and CD8+ OT-I cells were engrafted into wild-type, Cd80/86−/− or Ccr7−/− mice 1 day prior (OVA/CpG) (red) or (BSA/CpG) (blue) NP immunization (s.c.) into mouse right flank. Contralateral non-immunized left side served as control (gray). **(B)** CFSE dilution in engrafted OT-II cells (gated as CD45.1+ TCRβ+ CD4+) was analyzed in skin-draining LN 3 days p.i. **(C)** (Left) Histobar graph shows percentages of engrafted splenic OT-II cells 5 days after immunization (OVA) (gray) or (OVA/CpG) (red) NP. (Right) IFN-γ and IL-17A secretion measured by intracellular staining in engrafted splenic OT-II cells 5 days p.i. with (OVA CpG) NP. Cells were *in vitro* restimulated O.N. with PMA/Ionomycin and OVA peptide (323–339). **(D)** Antigen-presentation ability of *in vivo* antigen-loaded DC was measured *in vitro* by the CFSE dilution of CD4+ OT-II cells after 3 days of co-culture with DC. NP+ DC and NP- DC were sorted from LN 16 h p.i. with (OVA/CpG) or (BSA/CpG) NP and co-culture with CFSE-labeled CD4+ OT-II cells in a 1 DC:4 T cells ratio. Results are shown as mean ± SD and are representative of more than 3 independent experiments, *n* = 8–12, individual mice. ^*^p ≤ 0.05, ^****^p ≤ 0.0001.

NP immunization induced robust antigen specific proliferation of CD4^+^ and CD8^+^ T cells, as indicated by label dilution of CFSE-treated grafts (Figures 2A,B, Supplementary Figure 2A). T cell responses depended on costimulation as they were abrogated in T cell-engrafted CD80/CD86 double deficient mice (Figure 2B, Supplementary Figure 2A). Moreover, CD4^+^ and CD8^+^ T cell expansion was also reduced in CCR7 KO animals suggesting requirement of DC migration for efficient priming (Figure 2B, Supplementary Figure 2A). Immunization with NP harboring both CpG and OVA antigen resulted in robust T_H_1 polarization of the engrafted CD4^+^ OT-II cells, as indicated by IFN-γ secretion at day 5 p.i. (Figure 2C). Also engrafted CD8^+^ OT-I cells displayed IFN-γ secretion, as they differentiated into mature CTL (Supplementary Figure 2B).

To confirm that NP-carrying DC are directly responsible for antigen presentation and T cell stimulation early after immunization, NP^+^ and NP^−^ DC were retrieved 16 h after immunization for co-culture with CFSE-labeled OT-II cells (DC:T ratio 1:4). T cell proliferation, as indicated by CFSE dilution, was in absence of exogenous peptide addition exclusively observed with NP^+^ DC isolated from (OVA/CpG) NP challenged mice, but not with NP^−^ DC isolated from the same LNs, in presence of (BSA/CpG) NP^+^ DC or DC isolated from non-immunized controls (Figure 2D, Supplementary Figure 2C). Collectively, these data establish that (OVA/CpG) NP immunization results in “mature” NP-carrying DC that induce a robust antigen-specific T cell response associated with T_H_1 polarization.

### Transcriptome Analysis of Antigen-Presenting DC

**Figure 3:**
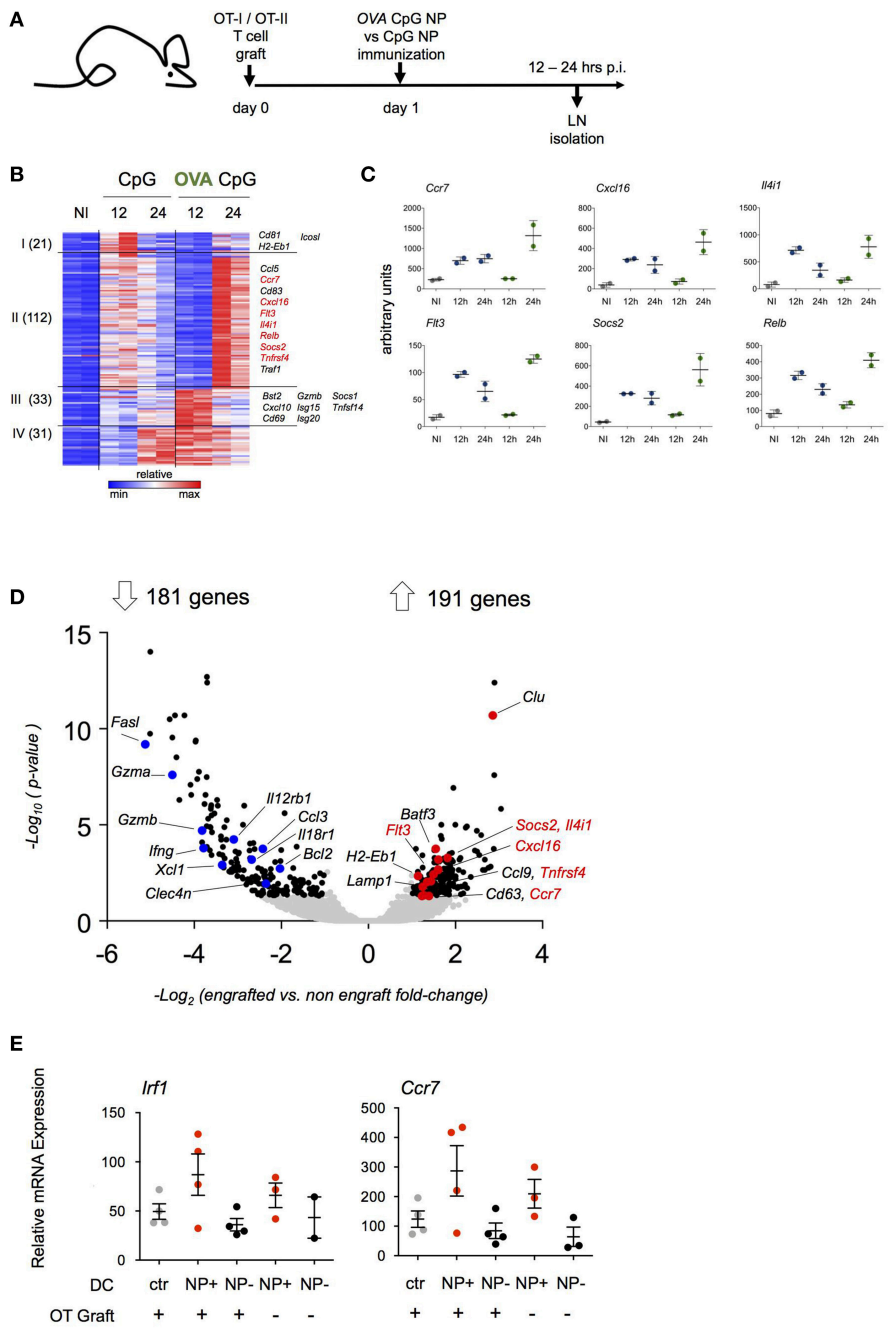
Transcriptome analysis of antigen-carrying DC. **(A)** Schematic experimental protocol. NP-carrying DC isolated from skin-draining lymph nodes were sorted for RNAseq analysis 12 and 24 h p.i. Immunization with (OVA/CpG) NP was compared to (CpG) NP to extrapolate antigen signature from the adjuvant effect. *n* = 2, each sample is a pool of 2 mice. **(B)** Heatmap of 197 genes differentially expressed between two conditions (OVA/CpG vs. CpG, fold-change>2 and adj-*p* < 0.05) separated into 4 clusters by unbiased k-means clustering. *n* = 2, each sample is a pool of 2 mice. **(C)** Graphs showing normalized reads of genes in **(B)**. Each dot represents a pool of 2 mice, *n* = 2, line represents mean ± SD. **(D)** Activation of LN DC after NP immunization was tested in absence of prior engraftment of OVA responding cells. NP-carrying DC isolated from skin-draining LN were facs-sorted for transcriptional RNAseq analysis 12 h p.i. with (OVA/CpG) NP. Prior immunization only a group of mice was engrafted with CD4+ OT-II and CD8+ OT-I cells. Volcano plot shows distribution of 372 differentially expressed genes (black) out of 8754 genes (gray) between the two experimental protocols (graft vs. no graft fold-change>2 and adj.*p*-value < 0.05). *n* = 2, each sample is a pool of 2 mice. **(E)** *Irf1* and *Ccr7* mRNA expression levels measured by real-time qPCR in skin-draining LN DC 12 h upon (OVA/CpG) NP immunization. DC were sorted accordingly to the absence (NP- DC, black) or the uptake (NP+DC, red) of rhodamine-labeled NP in the immunized samples. DC from non-immunized mice served as control (gray). Results are shown as mean ± SD and are representative of two independent experiments. *n* = 4, each sample is a pool of two mice.

The aim of this study was to define the DC responses to a cognate T cell encounter. The latter could be masked by responses to the adjuvants. We therefore decided to compare two NP formulations: NP that entrapped CpG adjuvant (CpG NP) and NP that entrapped CpG and were adsorbed OVA (OVA/CpG NP). Prior to the immunizations, mice were engrafted with OT-I and OT-II cells and then hock challenged with CpG NP or OVA/CpG NP (Figure 3A).

DC harboring NP (NP^+^ DC) were retrieved from inguinal and popliteal LNs and RNAseq analysis was performed (Supplementary Figure 3A). As expected, NP immunizations resulted in a robust DC response compared to cells isolated from non-immunized LNs. DC isolated from LNs of (OVA/CpG) NP-challenged mice did not show exclusively induced genes that were not observed following CpG NP immunization (Supplementary Figure 3B). Rather, immunization with the OVA-coated NP resulted in distinct kinetics of gene expression (Figure 3B). One hundred and thirty-three of the total 197 induced genes (cluster I and II) displayed a delayed induction when NP carried the antigen recognized by the T cell graft; 122 of these genes were induced more persistent and robust (cluster II). The latter list of genes included *Ccl5, Ccr7, Socs2, RelB*, and *Flt3* (Figure 3C). Sixty-four genes (cluster III and IV) displayed a more robust early induction in OVA/CpG NP^+^ DC. This included *Isg15, Cxcl10* and *Tnfsf14*.

The above suggested that the transcriptome of NP^+^ OVA peptide-presenting DC that encountered antigen-specific T cells might differ from DC that had no T cell encounter. To address this aspect in a different way, we next compared NP^+^ DC isolated from mice that were immunized with (OVA/CpG) NP with or without prior transfer of OVA-specific T cells. In naïve C57BL/6 mice the frequency of OVA peptide-presenting DC that encounter an antigen specific CD4^+^ or CD8^+^ T cell is negligible, given that the number of antigen specific T cells in the naïve repertoire has been estimated to comprise 100–200 cells (33–35); in contrast, in animals that received the OT-I and OT-II T cell graft, any such DC would be expected to have immediate cognate T cell contact. When retrieved from the LNs 12 h post immunization, NP^+^ DC isolated from T cell engrafted and non-engrafted mice displayed 372 significantly differentially expressed genes out of a total of 8,754 genes (Figure 3D). 191 transcripts were preferentially upregulated in NP^+^ DC that encountered antigen-specific T cells. Interestingly and supporting the notion that they indeed might indicate a cognate T cell encounter, this included genes that had shown the distinct kinetics in the above experiment (Figure 3D), such as *Ccr7, Cxcl16, Flt3, Il4i1, Socs2*, and *Tnfrsf4*. Preferential induction of *Irf1* and *Ccr7* in presence of OVA-specific T cells was confirmed by RT-PCR (Figure 3E). Collectively, these data suggest that the expression profile of antigen presenting DC in the immunized LNs is affected by an encounter with antigen specific CD4^+^ or CD8^+^ T cells.

### Cognate T Cell Interactions Affect the DC Transcriptome

**Figure 4:**
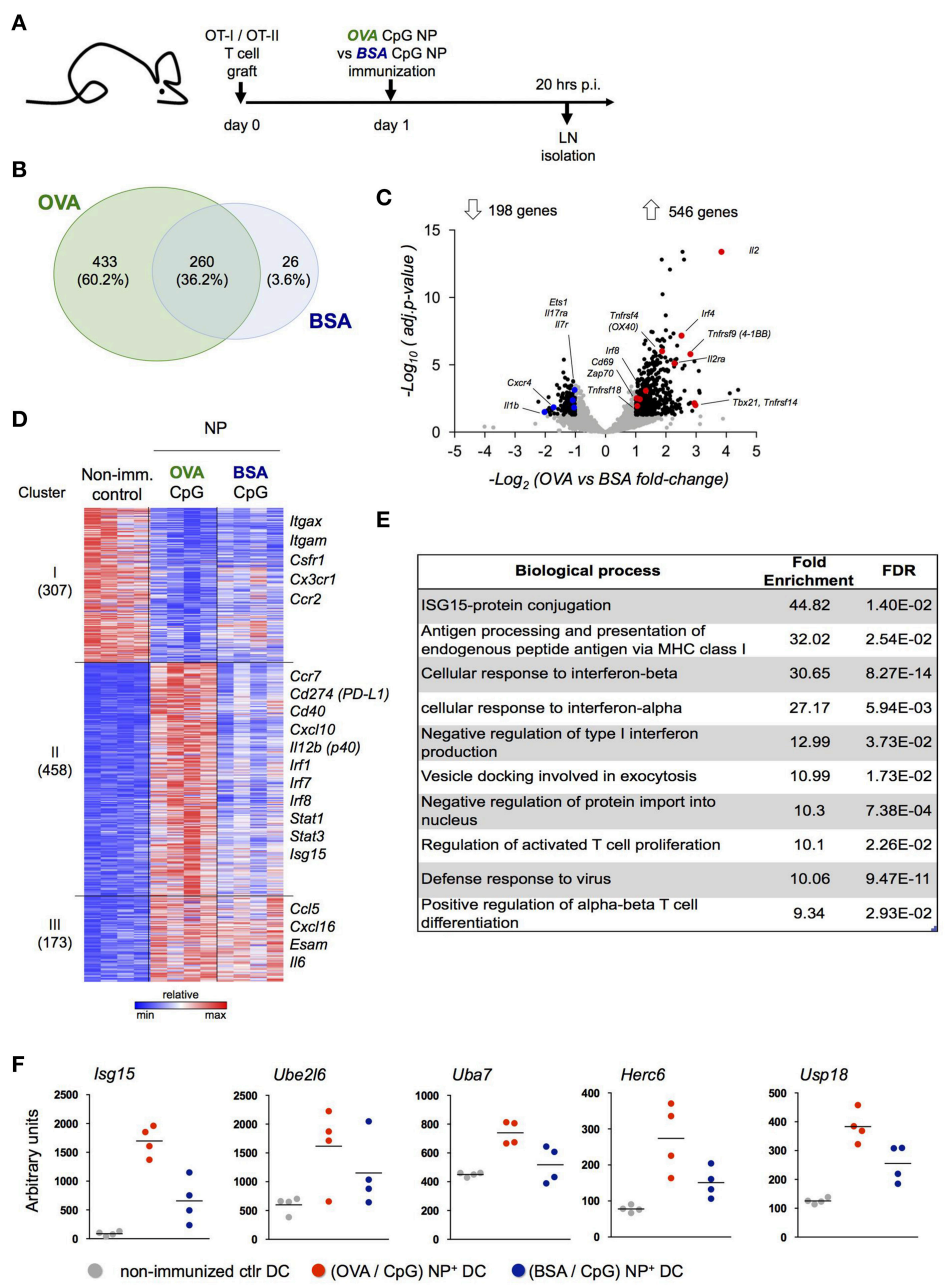
Definition of the DC signature indicating cognate interaction with antigen-specific T cells. **(A)** Prior immunization with (OVA/CpG) NP or (BSA/CpG) NP, mice were engrafted with OVA-responding T cells. Rhodamine-labeled NP+ DC and CD4^+^ OT-II cells were sorted 20 h p.i. for transcriptional RNAseq analysis. **(B)** Genes differentially expressed in NP^+^ DC (vs. NI control DC, fold-change>1.8 & adj.*P*-value < 0.05); overlap of the two immunization protocols is shown in the Venn diagram. **(C)** In CD4^+^ OT-II cells, distribution of 744 differentially expressed genes (black) from a total of 7,830 genes (gray) in the immunization protocols comparison is shown by the volcano plot (OVA vs. BSA fold-change>1.8 & adj.*p*-value < 0.05). *n* = 3, each sample is a pool of 2 mice. **(D)** 938 differentially expressed genes (OVA/CpG vs. NI, BSA/CpG vs. NI or OVA/CpG vs. BSA/CpG adj-*p* < 0.01) of NP+DC were separated into 5 clusters by unbiased k-means clustering. *n* = 4, each sample is a pool of 2 mice. **(E)** Enriched GO terms among 458 differentially expressed genes included **(D)**, cluster II. **(F)** Normalized reads of the genes involved in the Isg15 protein-conjugation pathway included in **(D)**, cluster II. Each dot represents a pool of 2 mice, *n* = 4, line represents mean.

Our data suggest that peptide-presenting DC receive signals from synapse-forming antigen specific CD4^+^ or CD8^+^ T cells that alter the DC expression profile. To further test this notion, we performed a third complementary approach. Specifically, we hock immunized T cell-engrafted mice with NP harboring CpG and coated with OVA (OVA/CpG NP) or the control antigen BSA (BSA/CpG NP) (Figure 4A) and isolated NP^+^ DC for RNAseq (Supplementary Figure 4A). When compared to control DC retrieved from non-immunized LNs (NI), NP^+^ DC isolated 20 h post challenge from (OVA/CpG) NP and (BSA/CpG) NP immunized animals displayed 693 and 286 differentially expressed genes, respectively (vs. NI control DC, fold-change>1.8 & adj. *p* < 0.05) (Figure 4B). The large majority of the transcripts observed in (BSA/CpG) NP^+^ DC (90%) were shared with NP^+^ DC isolated from animals immunized with (OVA/CpG) NP, likely reflecting the response to CpG. In addition though, (OVA/CpG) NP^+^ DC displayed altered expression of 433 genes, as compared to NI DC, that were non-significantly changed in (BSA/CpG) NP^+^ DC over the NI sample, suggesting a relation to the encounter of OVA-specific T cells (Figure 4B).

To ensure that 20 h post immunization antigen specific T cells had encountered DC and themselves responded, we performed a parallel RNAseq analysis of OT-II cells isolated from the LNs of the same mice (Figure 4C, Supplementary Figure 4B). OT-II cells retrieved from (OVA/CpG) NP immunized mice displayed a robust response with 546 induced genes, as compared to OT-II cells isolated from (BSA/CpG) NP immunized mice (fold-change>1.8, adj.*p*-value < 0.05) (Figure 4C). The T cell response included genes important for T cell activation and survival (*Cd69, Il2, Il2ra*), costimulatory signal reception (*Tnfrsf4, Tnfrsf9, Tnfrsf14, Tnfrsf18*), TCR signaling (*Zap70*) and T_H_1 differentiation (*Tbx21*) (Figure 4C).

To further characterize the DC response to T cell encounter we next performed a pathway analysis. Unbiased *k*-means analysis of the 938 genes differentially expressed in NP^+^ DC, as compared to control DC (NI) (adj.*p*-value < 0.01), led to segregation into 3 clusters (Figure 4C). (OVA/CpG) NP-carrying DC differed from (BSA/CpG) NP-carrying DC with respect to induced expression of 458 genes (cluster II, Figure 4B). This cluster comprised markers of maturation and costimulation (*Ccr7, Cd40, Cd274*), transcription factors (*Irf1, Irf7, Irf8, Stat1, Stat3*), as well as the T_H_1 polarizing cytokine *Il12b*. Ingenuity Pathways Analysis of cluster II revealed CD40 as upstream regulator of this cluster, that could indicate engagement by CD40L expressed on activated T cells (Supplementary Table 1). Analysis for enriched gene ontology (GO) terms further supported the notion that cluster II comprises genes associated with a cognate T cell encounter. Specifically, GO analysis revealed signatures for antigen-processing and presentation, a response to type I interferon and T cell activation that were restricted to the OVA peptide-presenting DC genes (cluster II, Figure 4E). GO analysis further highlighted a signature related to Isg-15 protein conjugation. Indeed, (OVA/CpG) NP-carrying DC displayed up-regulation of the whole Isg15 pathway, including the ubiquitin-like molecule itself (*Isg15*), its activating E1 enzyme (*Uba7*), its conjugating E2 enzyme (*Ube2l6*), the E3 ligase (*Herc6*) and the related peptidase (*Usp18*) (Figure 4F).

**Figure 5:**
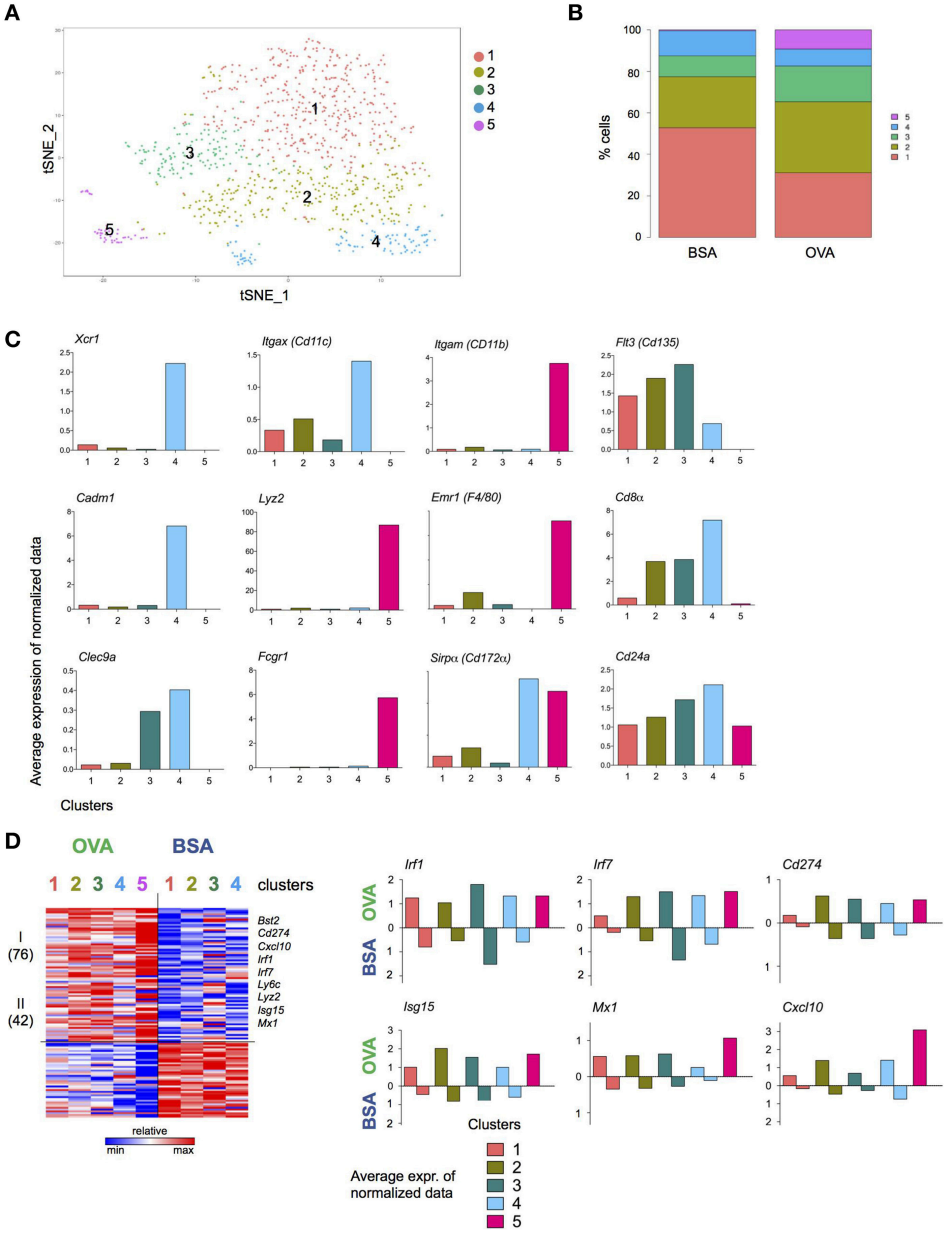
T cell interacting DC signature revealed by single cell transcriptome analysis. Experimental lineup is represented in Figure 4A. **(A)** 476 (OVA/CpG) and 496 (BSA/CpG) NP^+^ DC were analyzed and portioned into 5 clusters. **(B)** OVA and BSA carrying DC contribution to each cluster. **(C)** Examples of cDC1 and moDC genes is shown as the log average expression of normalized data in each cell cluster. **(D)** (Left) Heatmap represents Z scores of log average expression of normalized data of 118 differentially expressed genes (OVA vs. BSA) separated into 2 gene clusters by *k*-mean method. (Right) Average expression of normalized data for representative genes *Isg15, Irf1, Cxcl10* and *Mx1*. Gene expression of both OVA- and BSA-carrying DC is shown separated. Cluster is not shown due to lack of cells.

To gain further insights into the DC response to T cell encounter, as well as subpopulations among the NP^+^ DC, we complemented the bulk population analysis (Figure 4) with single cell transcriptomics (Figure 5). (OVA/CpG) or (BSA/CpG) NP^+^ DC were single-cell sorted into 384w-plates, barcoded and MARS-sequenced (36). Four hundred and seventy-six (OVA/CpG) NP^+^ DC and 496 (BSA/CpG) NP^+^ DC were analyzed and portioned into 5 cell clusters (Figure 5A, Supplementary Figure 5A). Cluster 1 to 4 were roughly equally represented in the DC populations isolated from (OVA/CpG) or (BSA/CpG) immunized LNs. In contrast, cluster 5 formed exclusively among (OVA/CpG) NP^+^ DC (Figure 5B). Clusters 1–3 likely include cDC2, as well as migratory and double-negative DC. Precise definition of these subsets remains challenging (6, 7, 9, 37) and might require deeper analysis with respect to cell numbers and sequencing coverage. Cluster 4 comprised cDC1 as indicated by the expression of *Xcr1, Cadm1*, and *Clec9a* that have emerged as markers for this DC subset (11) (Figure 5C).

Comparison of the highly variable genes expressed by (OVA/CpG) NP^+^ DC and (BSA/CpG) NP^+^ DC belonging to the same cell cluster 1, 2, and 3 showed largely overlapping gene expression patterns (Supplementary Figure 5B). cDC1, as represented by cell cluster 4, however displayed differential gene expression in OVA/NP^+^ DC and BSA/NP^+^ DC, with 201 upregulated and 192 downregulated genes (gene cluster II and V) (Supplementary Figure 5B). The list of higher expressed genes included *March6, Ubd, Ube2b*, and *Ubr3* (protein ubiquitination, gene cluster II). Lower expressed genes included *Xcr1* (genes cluster V).

Cell cluster 5 whose appearance was restricted to NP^+^ DC retrieved from (OVA/CpG) NP immunized mice comprised cells expressing monocyte-associated genes, such as *Emr1, Lyz2*, and *Fcgr1* (Figure 5C), suggesting that these cells were recruited to the challenged LNs. These cells also expressed higher levels of *Itgam* (CD11b)*, Emr1* and *Sirp*α (CD172α) and lower levels of *Flt3* (CD135), *Cd24a, Cd8*α, and *Itgax* (CD11c) than cDC of cell in clusters 1–4 (Figure 5C).

The list of 118 genes differentially expressed between all DC retrieved from OVA/CpG NP-and BSA/CpG NP-challenged LNs revealed a dichotomous pattern. Specifically, 72 genes were higher expressed by OVA/CpG NP—carrying DC in all cell clusters, albeit with some preferences, including the transcription factors *Irf1* and *Irf7*, the inhibitory receptor *Cd274*, as well as the T cell and monocyte chemoattractant *Cxcl10* (38) (Figure 5D). Expression of the latter might contribute to the recruitment of the monocyte-like cells that was restricted to the OVA/CpG NP challenge. The list also comprised *Isg15*, corroborating a possible link of the ubiquitin-like protein to cognate DC—T cell interactions.

Collectively, these results suggest the existence of a bi-directional crosstalk between DC and T cells to promote T_H_1 response that should be further defined.

## Discussion

DC trigger T cells through MHC peptide presentation and costimulation, and subsequently direct activated T cells toward distinct effector fates. Here we designed an experimental system to identify transcriptomic changes in LN DC, which resulted from cognate T cell encounter. A nanoparticulate system harboring a label, antigen (OVA), and adjuvant (CpG) (29, 39) was used to induce antigen-specific T_H_1 response in OVA-responding TCR transgenic CD4^+^ cells (OT-II). NP were efficiently ingested by MHC II-expressing cells and induced robust antigen-specific T cell responses with minimal bystander activation. Given that this antigen-specific T cell response was abrogated in CD80/CD86 deficient mice unable to deliver costimulation, we concluded that our NP immunization challenge relied on the antigen presentation process for induction of T cells, excluding anergic activation (40).

Whether the interactions with cognate T cells in turn license the DC to acquire polarization potential has remained unclear. T cell interactions with mature DC occur with different degrees of T cell receptor engagement and allow lasting cell surface contact between DC and T cells (41, 42), that could play in favor of a bi-directional cross-talk between the two cell types involved. Indeed earlier reports proposed that CD40 and LTβR signaling provided by T cells cooperate to provide full conditioning of DC during antigen presentation (43) and immune synapsis signaling could protect DC from apoptosis (44). By manipulating our experimental system, we explored the bi-directional cross-talk between DC and T cells during immune synapsis. We found that cognate interaction modulate the transcriptomic changes DC undergo following adjuvants challenge and NP uptake mostly in terms of timing and intensity/robustness. A first series of experiment aimed to define the “masking” effect of the adjuvant CpG and revealed that (antigen/adjuvant) NP led to a similar response as (adjuvant) NP in terms of transcriptome alterations, albeit with a distinct kinetics. Interestingly, removing the antigen from the challenge or the antigen-responding T cells from the experimental setup while keeping the (antigen/adjuvant) NP challenge led to similar transcriptomic alterations suggesting a signature associated with cognate interaction with T cells. The genes modulated in both experimental setups included *Ccr7, Cxcl16, Flt3, Il4i1, Socs2*, and *Tnfrsf4*. The results were further corroborated in a complementary comparison of (antigen/adjuvant) NPs carrying different antigens (OVA or BSA). Here, cognate interaction was impeded by the inability of (BSA/CpG) NP-carrying DC to interact and present antigen or by the scarcity of OVA-responding T cells (33–35).

A module that seems particularly affected by the cognate T cell interaction was centered around ISG15, an ubiquitin-like protein with pleiotropic functions, induced by type I IFN (45). ISG15 is established as critical protein modifier during anti-viral responses with *Isg15*^−/−^ mice being more susceptible to infection (46–48). Conversely, the inactivation of ISG15-isopeptidase USP18, which results in accumulation of ISG15-conjugated substrates, increased viral resistance (48). Furthermore, emerging evidence suggests an extra-cellular role of secreted ISG15, in addition to its role as intra-cellular protein modifier. Secreted ISG15 was shown to engage the integrin CD11a on human NK cells and trigger, potentially in synergy with IL-12, IFNγ secretion (49). Likewise, release of cysteine-reactive free ISG15 was reported to induce cDC1 recruitment to the sites of *T. gondii* infection, and induce DC secretion of IL-1β production (50).

Finally, our single-cell RNAseq analysis revealed the recruitment of monocyte-derived DC upon cognate DC/T cell interactions. The population of monocyte-derived cells resembled earlier reported monocyte-derived DC, which were reported to contribute to T_H_1 responses by inducing IFNγ and IL-2 production in T cells (51). Although in that study recruitment of these cells was observed following LPS, direct CpG injection did not promote monocyte-derived DC recruitment to the LNs (probably due to low expression of TLR9 in monocytes) (51). Nevertheless, our experimental setup and analysis implies the engagement of an antigen specific recognition between DC and T cells, which cannot occur with the same frequencies upon direct CpG injection alone. It remains unclear why recruitment of monocyte-like cells seems restricted to the (OVA/CpG) NP challenge, although this could be related to enhanced chemokine expression by the other DC subsets including the established monocyte attractant CXCL10 (38). This supports the notion that cell recruitment in our model might be related to signals released during cognate DC/T cell interactions. Our study suggests a potential role of the ISG15 module in the establishment of T_H_1 responses, which should be substantiated by further experimentation, including the definition of intra- and extra-cellular modes of ISG15 action.

## Materials and Methods

### Mice

Mice aged 8–16 weeks old were used for experiments. The strains used included C57BL/6 wild-type purchased from Envigo (Harlan), *Ccr7*^−/−^ (B6.129P2(C)-*Ccr7*^*tm*1*Rfor*^/J, #006621) (52), *Cd80/86*^−/−^ (B6.129S4-*Cd80*^*tm*1*Shr*^ *Cd86*^*tm*2*Shr*^/J, #003610) (40), OT-I (C57BL/6-Tg(TcraTcrb)1100Mjb/J #003831) (53), OT-II (B6.Cg-Tg(TcraTcrb)425Cbn/J, #004194) (54) and CD45.1 (B6.SJL-*Ptprc*^*a*^ *Pepc*^*b*^/BoyJ, #002914) purchased by Jackson Laboratory and bred in the Weizmann Institute of Science.

Mice were maintained on a 12 h light/dark cycle, and food and water were provided *ad libitum*. All animals were on a C57BL/6JOlaHsd background, maintained in specific-pathogen-free conditions and handled according to protocols approved by the Weizmann Institute Animal Care Committee (IACUC), as per international guidelines.

### Nanoparticle Preparation

Biodegradable polymeric nanoparticles (NP) were prepared by double emulsion solvent evaporation technique (w/o/w), as previously reported (29). Briefly, the aqueous solution was added to the polymer (aliphatic-polyester poly(lactic-co-glycolic acid), PLGA, Sigma-Aldrich) previously dissolved in the organic solvent dichloromethane (DCM). The single emulsion (o/w) was formed using an ultrasonic processor (Sonifier Vibracell VC 375, Sonics & Materials Inc.) at 70W for 15 s. Adjuvant CpG (ODN 1826, TCCATGACGTTCCTGACGTT, Microsyth) was dissolved in the internal aqueous solution (IP). Fluorescent NP were formulated by replacing 50 μL of organic polymer solution with Rhodamine-6G (Sigma-Aldrich) solution (2 mg/mL) in DCM. A 2% (w/v) polyvinyl alcohol (PVA, Sigma-Aldrich) solution was added to the (o/w) primary emulsion, and a second sonication was performed under the same conditions. The double emulsion (w/o/w) was added dropwise to the external surfactant phase (EP) with 0.3% (w/v) PVA, and stirred at 37°C for 1 h. The NP suspension was washed twice with ultrapure water by centrifugation (22,000 x g, 45 min, 4°C; Sorvall Lynx 4000 centrifuge, Thermo Fisher). Ovalbumin (OVA, 1 mg/ml, Sigma-Aldrich) or bovine serum albumin (BSA, 1 mg/ml, Sigma-Aldrich) were adsorbed onto NP after 1 h of incubation at room temperature. NP were washed with ultrapure water and centrifuged for 20 min (22,000 x g, 4°C; Sorvall Lynx 4000 centrifuge, Thermo Fisher). Final pellet of NP was resuspended in phosphate buffered saline (PBS) pH 7.4 and kept at 4°C.

### Immunization Protocol

One day prior immunization mice were engrafted with a mixture of CD4^+^ OT-II and CD8^+^ OT-I cells, unless stated otherwise. Spleens were harvested from OT-II and OT-I mice, cell suspensions were obtained by glass-teasing the tissues, filtered through a 150 μm sieve and then incubated with anti-mouse CD4 or CD8 microbeads (Miltenyi), respectively, for 15 min at 4°C. CD4^+^ OT-II and CD8^+^ OT-I cells were positively enriched according to Magnetic Activated Cell Sorting (MACS) protocol (Miltenyi). For DC analysis or sorting experiments, 5 × 10^5^ CD4^+^ OT-II and 2 × 10^5^ CD8^+^ OT-I cells were injected intra venously (i.v.) into recipient mice. For T proliferation and differentiation assays, 1 × 10^6^ CD4^+^ OT-II and 4 × 10^6^ CD8^+^ OT-I cells were injected i.v. into recipient mice. Sixteen to twenty-four hours of after OT graft, mice received sub-cutaneous (s.c.) hock immunization (55). NP were resuspended in PBS, 50 μl of NP solution were injected in each flank of the mouse, unless stated otherwise. As previously described (29), the volume of NP used per injection carry 20 μg of OVA and 10 μg CpG.

### Multispectral Imaging Flow Cytometry Analysis

Cells were imaged using Multispectral Imaging Flow Cytometry (ImageStreamX markII flow cytometer; Amnis Corp, part of EMD Millipore, Seattle, WA). A 60x magnification was used for all analyzed samples. At least 30,000 cells were collected for each sample. Data were analyzed using a dedicated image analysis software (IDEAS 6.2; Amnis Corp). Images were compensated for fluorescent dye overlap by using single-stain controls. Cells were gated for single cells using the area and aspect ratio features, and for focused cells using the Gradient RMS feature (56). Cells were further gated using a bivariate plot for circularity (the degree of the mask's deviation from a circle) based on the Object mask (a segmentation mask that creates a tight fit on the cell morphology) and intensity of the side scatter channel (illuminated by the 785 nm laser and collected in channel 12). Particle internalization was calculated by the Internalization feature, i.e., the ratio of the intensity inside the cell to the intensity of the entire cell, mapped to a log scale. To define the internal mask for the cell, the object mask of the bright field image was eroded by 5 pixels.

### Flow Cytometry and Cell Sorting

Inguinal and popliteal lymph node were harvested and 12, 20, or 24 h post-immunization (p.i.), incubated 15 min with collagenase D (Roche) at 37°C and mechanically disrupted by glass-teasing. Cell suspensions were filtered through a 150 μm sieve and stain with biotinylated anti-mouse antibodies for lineage depletion (TCRβ, CD3ε, CD19, B220, NK1.1, Ly6G, PDCA-1) (Biolegend) for 20 min on ice. After washing, cells were incubated with anti-biotin microbeads (Miltenyi) for 15 min at 4°C. DC fraction enrichment was obtained by MACS negative selection (Miltenyi). After spin down, cells were stained with directly conjugated antibodies (CD45, CD11c, CD11b, I-Ab, lineage). Cells were acquired with LSR II Fortessa (BD) for analysis or facs-forted using FACSAria Fusion (BD). FlowJo (version 10, TreeStar) was used for post-acquisition analysis of the data.

### Quantitative Real-Time PCR

RNA was isolated from 5,000 to 10,000 DC previously facs-sorted into 40 μl of lysis buffer (Life Technologies). Dynabeads mRNA Direct Purification Kit (Life Technologies) was used following manufacturer's guidelines. RNA was reverse transcribed with High Capacity cDNA Transcription Kit (Applied Biosystems). PCR were performed with Platinum SYBR Green qPCR SuperMix (Life Technologies) and QuantStudio 6 Flex (Applied Biosystems). Quantification of the PCR signals of each sample was performed by comparing the cycle threshold values (Ct), in duplicate, of the gene of interest with the Ct values of the TBP housekeeping gene.

Primers used in the research: *Il6* Fwd 5′-CCTCTCTGCAAGAGACTTCCAT-3′, *Il6* Rev 5′-ACAGGTCTGTTGGGAGTGGT-3′, *Il12a* (p35) Fwd 5'-GCCACCCTTGCCCTCCTAA-3', *Il12a* (p35) Rev 5'-GGTTTGGTCCCGTGTGATGTC-3', *Irf1* Fwd 5′-GTTGTGCCATGAACTCCCTG-3′, *Irf1* Rev 5'-TGGACTTTCTCTCTTTCCTCTGG-3′, *Ccr7* Fwd 5′-CTCCTTGTCATTTTCCAGGTGTG-3′, *Ccr7* Rev 5′-GGCAGGAACCAGGCCTTAAA-3′, *Tbp* 5′-GAAGCTGCGGTACAATTCCAG-3′, *Tbp* Rev 5'-CCCCTTGTACCCTTCACCAAT-3′.

### T Cell Proliferation Assay

CD4^+^ OT-II and CD8^+^ OT-I cells were isolated as previously described. Cells were incubated at the concentration of 10 × 10^6^ cells/ml with carboxyfluorescein succinimidyl ester (CFSE, Biolegend) in PBS for 8 at room temperature in the dark. Equal volume of fetal bovine serum was added and cells were incubated for 5 min on ice protected from the light. After two washes with PBS, cells were counted and a mixture of 1 × 10^6^ CD4^+^ OT-II and 4 × 10^5^ CD8^+^ OT-I cells was injected i.v. into recipient wild-type, Ccr7^−/−^ or Cd80/86^−/−^ mice. On the following day, mice were immunized s.c. into the right flank with 50 μl of (OVA/CpG) or (BSA/CpG) NP solution. Left flank served as non-immunized control. Inguinal and popliteal LN were excised 3 days p.i. and analyzed by flow cytometry.

### T Cells Differentiation Assay

CD4^+^ OT-II and CD8^+^ OT-I cells were isolated as previously described. A mixture of 1 × 10^6^ CD4^+^ OT-II and 4 × 10^5^ CD8^+^ OT-I cells was injected i.v. into recipient wild-type mice. One day later, mice were immunized s.c. with 50 μl of the indicated NP into both flanks. Spleens were harvested 5 days p.i. Tissues were processed to yield a single cell suspension. After lysis of red blood cells, cell suspensions were washed and filtered through a 70 μm cell strainer. Cells were then incubated O.N. at 37°C, 5% CO_2_ in RPMI (Biological Industries) and stimulated with PMA/Ionomycin activation cocktail (1:2000, BioLegend), Brefeldin A (0.5 μg/ml, BioLegend) and OVA peptide (323-339, 20 μg/ml, GenScript). On the following day cells were stained for extracellular TCRβ, CD4, CD45.1, CD44 and intracellular IFNγ and IL-17A according to the guidelines of the Cytofix/perm kit (BD). Cells were analyzed by flow cytometry.

### *Ex vivo* Antigen Presentation Assay

Wild-type mice were immunized s.c. with (OVA/CpG) or (BSA/CpG) NP, non-immunized mice served as control. Inguinal and popliteal LN were excised 16 h p.i. and processed to yield single cell suspensions. As described above, after lineage depletion and staining, NP-carrying DC non-NP DC were facs-sorted according to their rhodamine signal, which indicated the uptake or not of NP by DC. Sorted DC were co-cultures with CFSE-labeled CD4^+^ OT-II cells in RPMI at 37°C, 5% CO_2_ in a ratio 1 DC: 4 T cells. When stated, OVA_323−339_ peptide was added at the concentration of 20 μg/ml. Cells were harvested after 3 days of co-culture and analyzed by flow cytometry.

### RNA Sequencing and Data Analysis

For bulk RNA-seq analysis, 5,000 DC were facs-sorted into 1.7 ml LoBind microtubes (Eppendorf) containing 40 μl of lysis buffer (Life Technologies). RNA was captured with Dynabeads mRNA Direct Purification Kit (Life Technologies) according to the manufacturer's instructions. The RNA-seq protocol for the generation of libraries is a derivation of MARS-seq (36). RNA-seq libraries were sequenced using Illumina NexSeq-500, raw data were mapped to the genome (NCBI37/mm9) using HISAT (version 0.1.6) (57), only reads with unique mapping were considered. Gene expression levels were calculated using HOMER software package (analyzeRepeats.pl rna mm9 -d < tagDir> -count exons -condenseGenes -strand + -raw) (58). Normalization and differential expression analysis were done using the DESeq2 R-package (Bioconductor, https://bioconductor.org/packages/release/bioc/html/DESeq2.html) (59). Differentially expressed genes were selected using a 2-fold change and *p*-value < 0.05 (Figures 3B,D), or a 1.8-fold-change and *p*-value < 0.05 (Figures 4B,C) or *p*-value < 0.01(Figure 4D) between at least two conditions. Gene expression matrix was clustered using a *k*-means algorithm (Matlab function kmeans) with correlation as the distance metric. Heat maps were generated using Genee software.

### Massively Parallel Single-Cell RNA-seq Library Preparation (MARS-seq)

As previously described by Jaitin et al. (36), cells were single-cell sorted into 384-well cell capture plates containing 2 μL of lysis solution and barcoded poly(T) reverse-transcription (RT) primers. Immediately after sorting, each plate was spun down to ensure cell immersion into the lysis solution, snap frozen on dry ice, and stored at −80°C until processed. mRNA from cell sorted into cell capture plates are converted into cDNA and pooled using an automated pipeline. The pooled sample is then linearly amplified by T7 *in vitro* transcription, and the resulting RNA is fragmented and converted into a sequencing-ready library by tagging the samples with pool barcodes and Illumina sequences during ligation, RT, and PCR. Each pool of cells was tested for library quality and concentration was assessed. All RNA-Seq libraries (pooled at equimolar concentration) were sequenced using Illumina NextSeq 500. Sequences were mapped to mouse genome (mm9), demultiplexed, and filtered as previously described (36), extracting a set of unique molecular identifiers (UMI) that define distinct transcripts in single cells for further processing. Mapping of reads was done using HISAT (version 0.1.6) (57); reads with multiple mapping positions were excluded. Reads were associated with genes if they were mapped to an exon, using the UCSC genome browser for reference. Exons of different genes that shared genomic position on the same strand were considered a single gene with a concatenated gene symbol.

### Filtering and Data Analysis of scRNA-Seq

Expression matrices from the four plates (two of BSA and two of OVA) were imported to a Seurat object (Seurat R Package (60); version 2.3.4), with minimal filtering criteria (min.cells = 5,min.genes = 300). Additional filtering of cells with high or low UMI or gene counts (below 5% value or above 95%) with the function FilterCells. Data was normalized and log transformed, scaled with regression [vars.to.regress = c(“nUMI”,“nGene”)]. Next 958 variable genes were detected [FindVariableGenes(mean.function = ExpMean, binning.method = “equal_width”,dispersion.function = LogVMR,x.low.cutoff = 0.1,num.bin = 40)]. Next, we performed a principal components analysis, and 8 significant principal components were used as input for FindClusters (resolution = 0.6). We used tSNE for two-dimensional visualization of the multi-dimensional dataset. Differential expression of the individual clusters was achieved using FindAllMarkers(only.pos = TRUE, min.pct = 0.25, logfc.threshold = 0.25). Gene Average expression per cluster was extracted with the function AverageExpression (uses normalized log values). One hundred and eighteen highly differentially expressed genes by (OVA/CpG) NP+ DC and (BSA/CpG) NP+ DC, were detected with fdr < = 0.05 using function FindMarkers with multiple statistical tests (MAST, poisson, negbinom, and bimod).

## Ethics Statement

All animals were on a C57BL/6JOlaHsd background, maintained in specific-pathogen-free conditions and handled according to protocols approved by the Weizmann Institute Animal Care Committee (IACUC), as per international guidelines.

## Author Contributions

CC designed and performed experiments. BB helped in experiments. EZ provided NP. AD performed additional experimentation. DJ, AG, ED, IA, LC-M, and DL helped with RNAseq and bioinformatic analysis. HF, K-PK, and IA critically advised. SJ conceived experiment and secured funding.

### Conflict of Interest Statement

The authors declare that the research was conducted in the absence of any commercial or financial relationships that could be construed as a potential conflict of interest.

## Supplementary Material

Supplementary Figure 1 **(A)** Uptake of NP in skin-draining LN by DC and non-DC. **(B)** Distribution of skin-draining LN XCR1^+^ cDC1 and CD11b^+^ cDC2 in non-immunized mice and upon OVA & CpG or BSA & CpG NP immunization. *n* = 6, individual mice.

Supplementary Figure 2 **(A)** Percentages of proliferating OT-I cells gated as CD45.1+ TCRβ+ CD4-. **(B)** (Upper) Histobar graph show percentages of engrafted splenic OT-I T cells (gated as CD45.1+ TCRβ+ CD4-) 5 days after immunization with BSA & CpG (blue), OVA (gray) or OVA & CpG (red) NP. (Lower) IFN-γ and IL-17A secretion measured by intracellular staining in engrafted splenic OT-I T cells 5 days p.i. with OVA & CpG NP. Cells were *in vitro* restimulated O.N. with PMA/Ionomycin and OVA peptide (323–339). **(C)** *In vitro* antigen-presentation ability of control DC sorted from non-immunized measured by CFSE dilution of CD4+ OT-II cells after 3 days of co-culture with DC. NP+ DC and NP- DC were co-culture with CFSE-labeled CD4+ OT-II cells in a 1 DC:4 T cells ratio in the presence of OVA_323−339_ peptide, when stated. Results are shown as mean ± SD and are representative of more than 3 independent experiments, *n* = 8–12, individual mice. ^***^*p* ≤ 0.001, ^****^*p* ≤ 0.0001.

Supplementary Figure 3 **(A)** Gating strategy for sorting of skin-draining LN derived NP+ DC for RNAseq of Figure 3. DC cells were defined and gated as CD45^+^ and lineage^−^ (lin: CD19, TCRβ, CD3e, NK1.1, Ly6G, Bst2). **(B)** Schematic experimental protocol is shown in Figure 3A. Heatmap of 953 genes differentially expressed between at least two conditions (OVA CpG vs. NI, CpG vs. NI or OVA CpG vs. CpG, Fold-change>2 & adj-*p* < 0.05) separated into 6 clusters by unbiased *k*-mean clustering.

Supplementary Figure 4 Experimental lineup is represented in Figure 4A. **(A)** Gating strategy for sorting of skin-draining LN derived DC. DC cells were defined and gated as CD45+, I-A^b^+ (MHC-II) and lineage- (lin: CD19, TCRβ, CD3e, NK1.1, Ly6G, Bst2). **(B)** OVA-responding T cells, which were engrafted into mice prior immunization, were gated and sorted as CD45.1+ TCRβ+ CD4+ from the lineage fraction of immunized LN 20 h p.i. for transcriptional RNAseq analysis.

Supplementary Figure 5 **(A)** Heatmap of the top 10 differentially expressed genes of each clusters (calculated on the overall average by all clusters). X-axis represents the gene expression by each single analyzed cell. Genes are listed on the Y-axis. **(B)** Heatmap shows 1,012 highly variable expressed genes separated into 5 clusters by unbiased k-mean clustering. Representative genes and GO analysis results for each cluster are indicated on the right side. The cell clustering was built on both (OVA/CpG) NP^+^ DC and (BSA/CpG) NP^+^ DC. This heatmap shows the expression patterns of DC carrying different antigen peptides (i.e., OVA or BSA). Although these DC belong to the same cell cluster, according to the modeling, some differences are to be noticed.

Supplementary Table 1 Nine hundred and thirty-eight of differentially expressed genes shown in the heatmap Figure 4D were analyzed for upstream regulators (Ingenuity Pathways Analysis).
