# Supplementary material for: DC Respond to Cognate T Cell Interaction in the Antigen-Challenged Lymph Node
Source: Front Immunol. 2019 Apr 25;10:863. doi: 10.3389/fimmu.2019.00863 (PMC6496461; doi:10.3389/fimmu.2019.00863)
Supplement: Supplementary Figure 1 — (A) Uptake of NP in skin-draining LN by DC and non-DC. (B) Distribution of skin-draining LN XCR1+ cDC1 and CD11b+ cDC2 in non-immunized mice and upon OVA & CpG or BSA & CpG NP immunization. n = 6, individual mice. [file Data_Sheet_1.pdf]

A

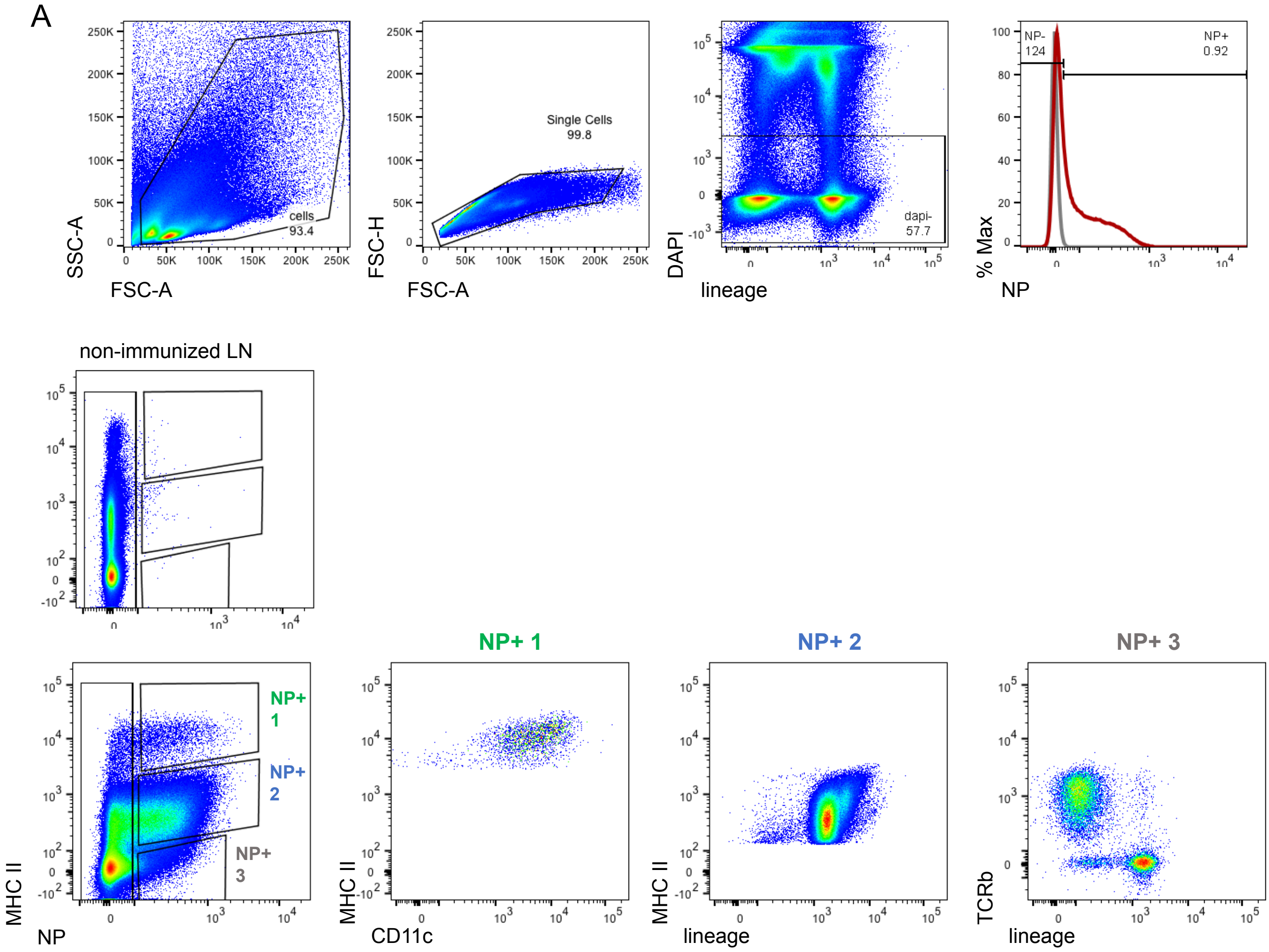


B

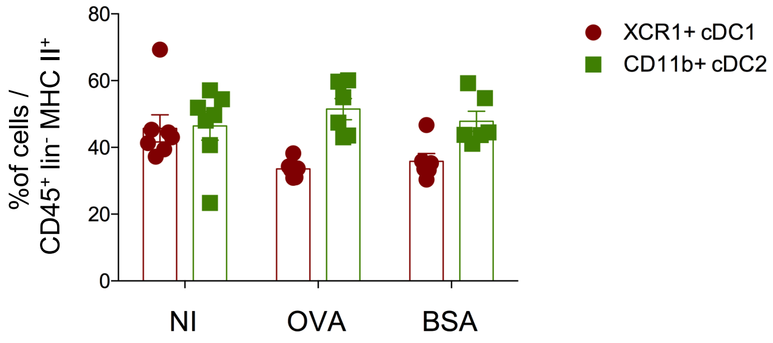


Suppl. Figure 1

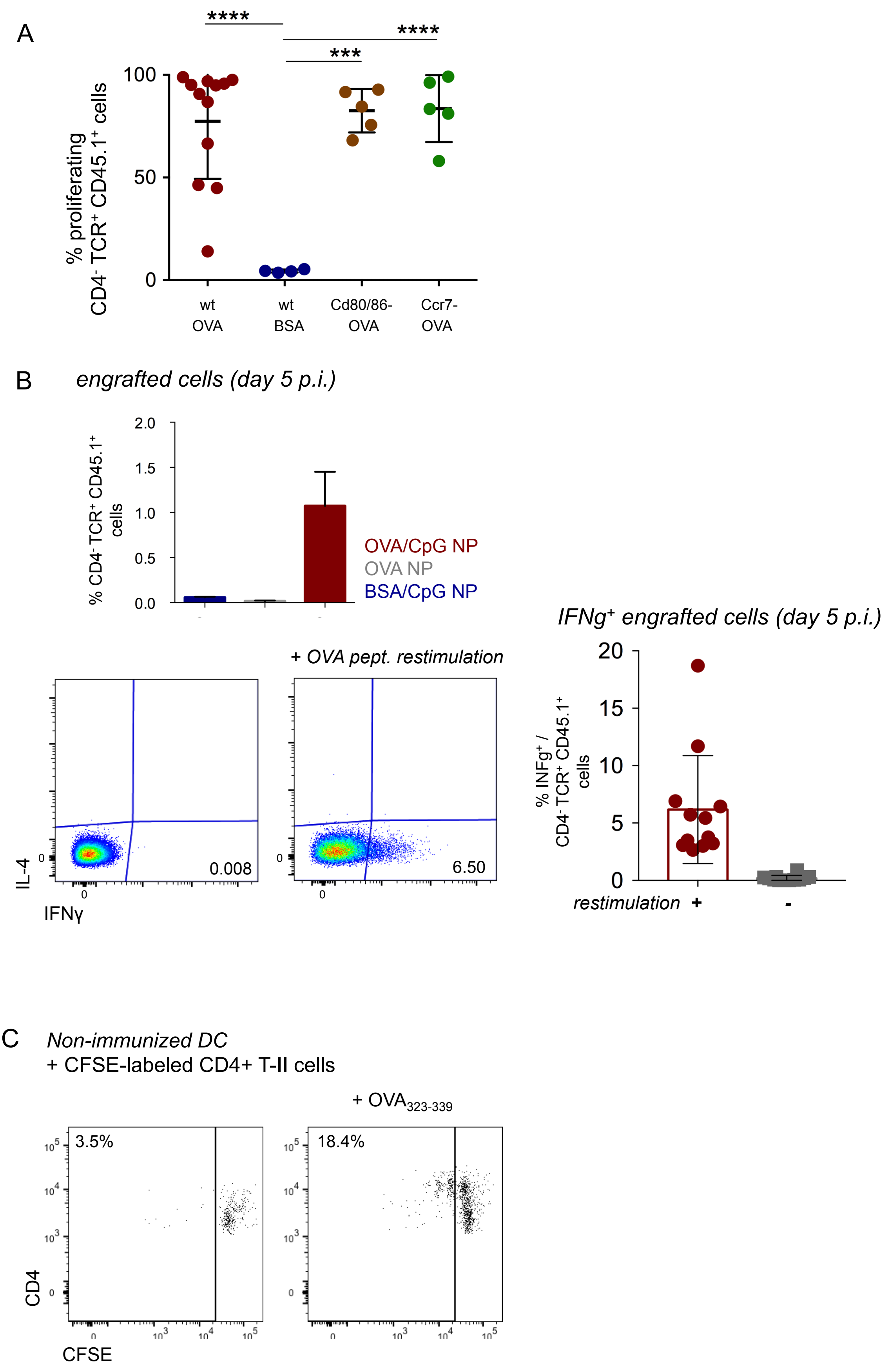


Suppl. Figure 2

A

Gating strategy (sorting from lin- fraction) – NP-carrying DC

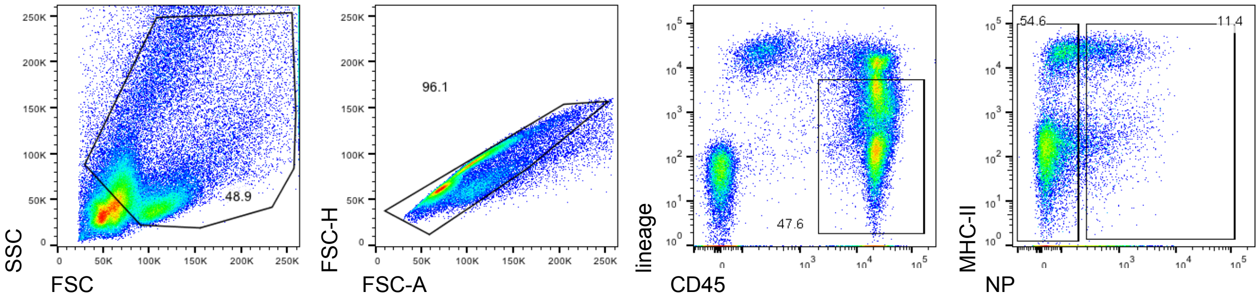


B

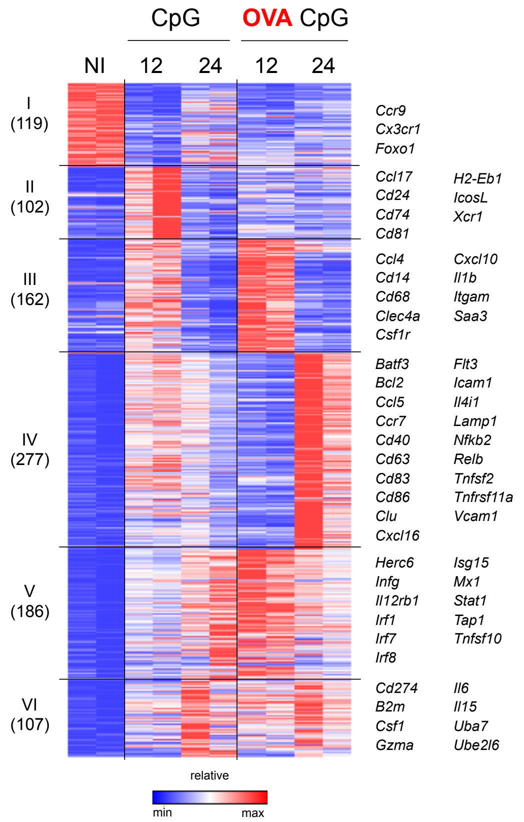


Suppl. Figure 3

A Gating strategy (sorting from lin- fraction) - DC

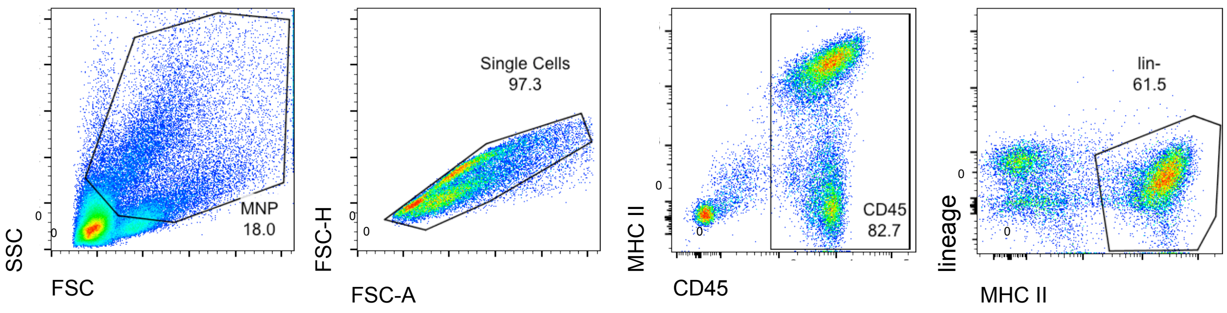


B Gating strategy (sorting from lin+ fraction) – CD4+ OT-II T cells

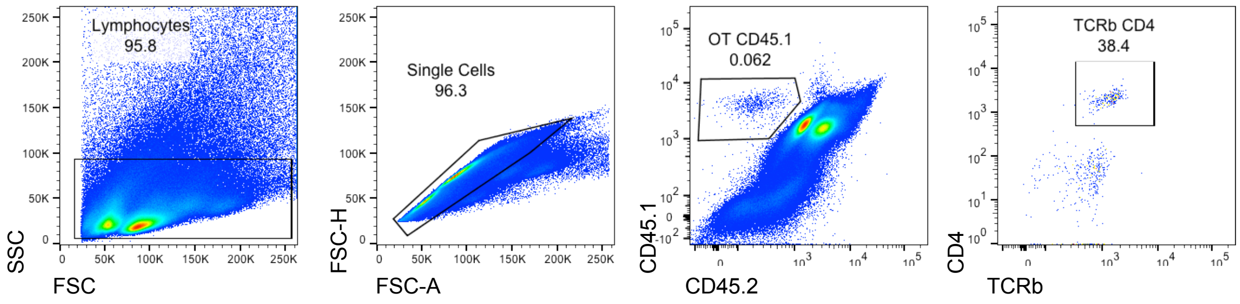


Suppl. Figure 4

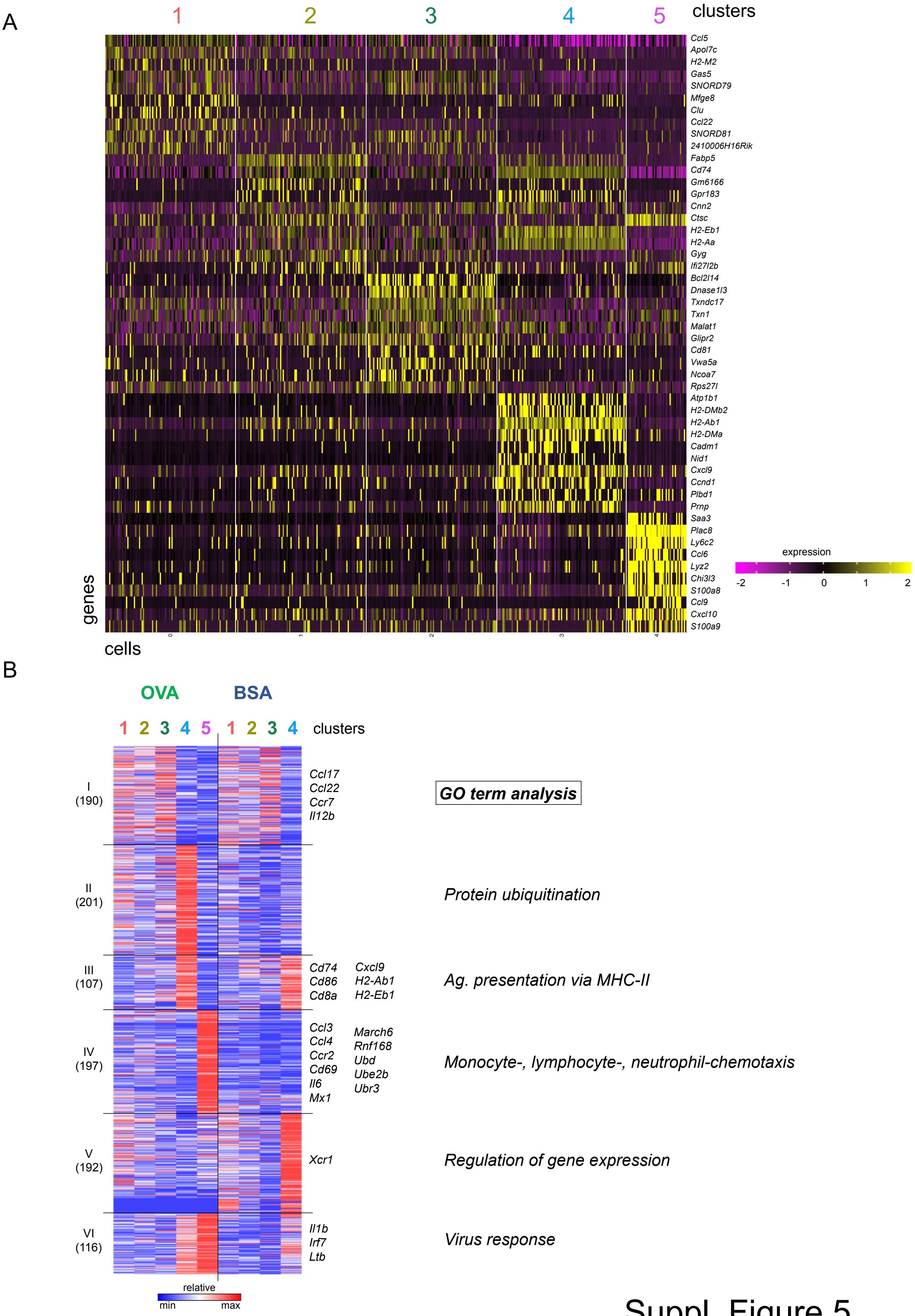


Suppl. Figure 5
