# Supplementary material for: DC Respond to Cognate T Cell Interaction in the Antigen-Challenged Lymph Node
Source: Front Immunol. 2019 Apr 25;10:863. doi: 10.3389/fimmu.2019.00863 (PMC6496461; doi:10.3389/fimmu.2019.00863)
Supplement: Supplementary Table 1 — Nine hundred and thirty-eight of differentially expressed genes shown in the heatmap Figure 4D were analyzed for upstream regulators (Ingenuity Pathways Analysis). [file Table_1.pdf]

Suppl. Table 1

Upstream regulators – Ingenuity Pathway Analysis

Upstream regulators	OVA	BSA	diff(OVA-BSA)
TLR3	2.42	0.00	2.42
TLR4	2.38	0.00	2.38
TP53	2.37	0.00	2.37
IRF1	2.22	0.00	2.22
RELA	2.19	0.00	2.19
RNASE2	2.18	0.00	2.18
TLR2	2.02	0.00	2.02
IL18	2.02	0.00	2.02
TBK1	1.98	0.00	1.98
TRPC1	1.98	0.00	1.98
NFKB1	1.98	0.00	1.98
WISP2	1.96	0.00	1.96
IRF7	1.95	0.00	1.95
TNF	2.69	0.77	1.92
TP63	1.67	0.00	1.67
CD40	1.14	-0.46	1.61
TGM2	3.06	1.49	1.57
PTGS2	1.52	0.00	1.52
P38 MAPK	1.51	0.00	1.51
IL1B	1.31	-0.20	1.51
IL15	1.50	0.00	1.50
NFkB (complex)	2.96	1.50	1.46
PI3K (family)	1.45	0.00	1.45
RNASE1	1.43	0.00	1.43
Hsp27	1.40	0.00	1.40
